# Empowering Prediction of Resting Energy Expenditure in Free-Living Settings by AI Tools: Application of a Population-Specific Equation from Saudi Arabia

**DOI:** 10.3390/nu18101618

**Published:** 2026-05-20

**Authors:** Yara Almuhtadi, Farah Mohammad, Jalal Al-Muhtadi, Ali Almajwal, Mahmoud M. A. Abulmeaty

**Affiliations:** 1 Community Health Sciences Department, College of Applied Medical Sciences, King Saud University, Riyadh 11433, Saudi Arabia; yalmuhtadi@ksu.edu.sa (Y.A.); aalmajwal@ksu.edu.sa (A.A.); 2 Center of Excellence in Information Assurance, King Saud University, Riyadh 12372, Saudi Arabia; fnazar@ieee.org (F.M.); jalal@ksu.edu.sa (J.A.-M.)

**Keywords:** resting energy expenditure, AI, ANN, XGBoost, free-living setting, population-specific equation

## Abstract

Background/Objectives: Traditional predictive equations derived from regression analyses exhibit varying degrees of accuracy in estimating resting energy expenditure (REE). AI models can increase the predictability of such equations, even for population-specific ones. This work aimed to improve the prediction of REE in a dataset of Saudi population-specific equations using suitable AI tools. Methods: The dataset from the previously published Saudi population-specific equation by Almajwal and Abulmeaty (AA) in 2019 was used to develop an artificial neural network (ANN)-based version to better predict REE in the adult population. Anthropometric and body composition parameters were used as proposed features. The proposed hybrid prediction model underwent an extensive two-stage, iterative training process. First, the Extreme Gradient Boosting (XGBoost) model is used to compute feature importance scores. Then, the most prominent features were identified and incorporated into the ANN model. These significant features were used to train the ANN model to capture nonlinear correlations among them and make accurate predictions. Subsequently, XGBoost and Convolutional Neural Network-Recurrent Neural Network (CNN-RNN) are used for their ability to provide a multi-layer abstraction of complex input data. Results: A total of 423 participants (208 male, 215 female) were divided into three non-overlapping sets: training (295, 70%), validation (64, 15%), and testing (64, 15%). The ANN model, combined with XGBoost, helped us to develop two equations: AA_ANN1= 2.47 × BMI + 11.9 × AdjBW + 962.5 and AA_ANN2 = 4.29 × age + 9.4 × fat mass + 15.71 × FFMI + 1289.3, where BMI is Body Mass Index (kg/m^2^), AdjBW is Adjusted Body Weight (kg), and FFMI is Fat Free Mass Index (kg/m^2^). The AA_ANN1 presented a Root Mean Square Error (RMSE) of 215 and an accuracy of 66.2%, whereas AA_ANN2 presented a lower RMSE of 193 and a higher accuracy of 71.4%. The ANN model was trained on the top 10 features ranked by XGBoost, achieving an average accuracy of 90.2%. Conclusions: The two new predictive equations, developed using an ANN combined with XGBoost, significantly improved REE prediction accuracy to 90.2%, achieved only with the full ANN model. Future external validation in an independent cohort is essential before clinical application of these equations.

## 1. Introduction

In the context of rising global obesity rates and non-communicable diseases (NCDs) [1], it is crucial to accurately estimate the nutritional needs of individuals and populations. Resting energy expenditure (REE) is a prerequisite for estimating energy expenditure and nutritional requirements among individuals [2]. REE can be measured accurately using Indirect Calorimetry (IC) or estimated using predictive equations that consider variables such as age, gender, weight, height, activity level, and stress [3,4].

The most used predictive equations for estimating REE include the Harris–Benedict equation [3], Schofield [5], and Mifflin-St Jeor [6] equations. The validity of these equations in Arabic or Saudi populations has been called into question because they were developed predominantly in American or European populations, which exhibit different body compositions than those of other demographic groups [7]. Furthermore, these equations fail to account for genetic variation [8] and ethnicity [8,9] within the same American population. To address these shortcomings, Almajwal and Abulmaety [7] developed a population-specific predictive equation for REE prediction tailored to the Saudi population, including both young and middle-aged males and females across various body mass index categories. The equation was developed using stepwise linear regression analysis of body composition and anthropometric parameters with measured REE using IC [7].

The regression-based equations have limited accuracy for estimating REE in individuals, which may lead to under- or overestimation of calorie requirements and health implications, such as obesity or malnutrition [10]. Therefore, accurate measurement of REE is critical for effective nutritional prescription, the development of dietary guidelines, and the design of intervention programs to tackle obesity and NCDs. Awareness of the potential for both underreporting and overreporting highlights the need for improved methods and practices in measuring energy expenditure. Artificial neural networks (ANNs) can provide a methodological improvement for predictive models, especially in the era of the artificial intelligence (AI) revolution. Previous research has demonstrated that artificial neural networks (ANNs) are a precise method for estimating REE in obese adults [11] and children [10]. Consequently, this study aims to develop an ANN model to improve predictions of REE in the Saudi population. Additionally, we seek to compare the accuracy of ANN-derived equations with that of traditional predictive equations.

A systematic study has been conducted to compare ANN predictive models with conventional predictive equations. In this respect, ANN was trained on a specific dataset to assess the effects of anthropometric and metabolic factors on its predictive performance. The proposed approach is a lightweight ANN algorithm combined with XGBoost, incorporating data collected by wearable electronic sensors.

## 2. Materials and Methods

### 2.1. Anthropometric and Body Composition Parameters

A consecutive sampling procedure was used to recruit all eligible participants who met the inclusion criteria for this study. Eligible participants included both male and female aged 18 to 60 years who were willing to undergo anthropometric measurements and body composition evaluation using standardized assessment techniques. Exclusion criteria included pregnant or lactating females and persons with any movement disabilities that would prevent accurate assessment. A total of 423 participants (208 male, 215 female) met all inclusion criteria and were included in the additional testing and analysis.

During the original project, participants were asked to complete a demographic questionnaire to obtain information on their age, sex, and pre-existing comorbid conditions. Body mass index (BMI) was calculated as weight (Wt) divided by height squared (kg/m^2^). Ideal body weight (IBW) was calculated as described in the original paper [7], and subsequently, adjusted body weight (AdjBW) was calculated as IBW + 1/4 (Wt-IBW). Waist-to-hip ratio (WHR) was calculated based on waist and hip circumferences.

Other anthropometric parameters included triceps skinfold thickness (TST), mid-arm circumference (MAC), and mid-arm muscle area (MAMA), which were calculated in millimeters, centimeters, and square centimeters, respectively. Percentage body fat was calculated based on bioelectric impedance analysis (BIA) (Tanita BC-418, Japan) measurements of body composition in addition to other BIA-based parameters, such as fat mass (FM), fat mass index (FMI), fat-free mass (FFM), fat-free mass index (FFMI), and total body water (TBW) were calculated as well. The detailed methods for obtaining anthropometric and body composition parameters were described in the original paper [7].

The reliability of anthropometric measurements was determined using the coefficient of reliability (R), defined as the proportion of total inter-individual variance not attributable to measurement error. When the coefficient of reliability approaches 1.0, especially at or above 0.95, it indicates that measurements are highly consistent and precise. General information on anthropometric data and major metabolic parameters for the participants is summarized in Table 1.

### 2.2. Resting Energy Expenditure (REE) Measurement

The REE was accurately measured in kilocalories per day (kcal/day) using the Indirect Calorimetry (IC) technique (Quark RMR, Cosmed, Italy), which employed the use of a ventilated canopy hood with a disposable antibacterial filter for assessing volumes of respiratory oxygen (VO_2_) and carbon dioxide (VCO_2_) as previously described in the original paper [7]. The VO_2_ and VCO_2_ were used to derive the Respiratory Quotient (RQ = VCO_2_/VO_2_), which allowed for the subsequent determination of the REE using the modified Weir’s equation, which specifically omitted the urinary nitrogen excretion component as per the reference [12]. A key aspect of ensuring the validity of the obtained results involved the achievement of the steady-state point, which involved the variation in the RQ values of <5% over a period of no less than five minutes, as well as the variation of the values for both the VO_2_ and minute ventilation of <10%. Results that failed to meet the steady-state point requirements or that were not considered for evaluation.

### 2.3. Prediction Equations

To facilitate comparison of the predictive accuracy of the updated equation, energy expenditure was also calculated using six widely accepted predictive equations. These equations specifically included two different versions of the Schofield equation—one using only weight as a predictive component [5] and another using both weight and height [5], the Oxford equation [13], the original Harris–Benedict equation [3], the Mifflin equation [6], the WHO equation [14], and the Saudi population specific equation by Almajwal-Abulmeaty equation AA_1 [7]. These various predictive formulas provided a framework for the value of the directly measured REE.

### 2.4. Proposed ANN Framework

The proposed hybrid prediction model underwent an extensive two-stage, iterative training process. First, the XGBoost model was used to compute feature importance scores by measuring how much each feature contributes to reducing the model’s loss. Based on those scores, the most prominent features were identified and incorporated into the ANN model. These significant features were used to train the ANN model to capture nonlinear correlations among them and make accurate predictions. This hybrid model efficiently combines the best features of XGBoost and ANN models, thereby increasing its predictive accuracy and manifold power.

The XGBoost [15] is a variant of Error Correction Learning models that uses a series of models, trained and frozen, to eliminate the prediction error generated by the previously trained models. It does so by adding the weighted gradient *g*(*x*), obtained through a loss function, to the prediction function *P*(*x*),

$$P^{t}\left(x\right)=P^{t-1}\left(x\right)+\alpha g^{t}(x)$$

where the prediction result after the *t*-*th* step, *F*(*x*), is expressed based on the prediction result of the previous (*t* − 1) step, with a weighted gradient component, *g*(*x*). Also, α is a learning rate parameter that dictates the step size, while *g*(*x*) denotes the computed gradient, which directs the learning process. XGBoost models are renowned for their ability to capture non-linear patterns in the attribute space. This strength is harnessed to validate the importance of attributes.

In particular, the ANN model [16] itself encompasses the design of a fully connected neural network specifically targeted to handle high-dimensional inputs and make high-quality predictions. The proposed neural networks were initiated with the Input Layer that receives the highly ranked feature set. This consists of three dense hidden layers with 128, 64, and 32 neurons, followed by an output layer. Each of these hidden layers first linearly transforms the input, then applies the ReLU activation function to provide the non-linearity essential to this model. To prevent overfitting and improve generalization to unseen data, dropout regularization is applied after every hidden layer by randomly deactivating neurons during each training iteration. Finally, it ends with the output layer, which uses the ReLU activation function to produce the final predicted output.

The choice to implement XGBoost for feature selection in combination with ANN for modeling was based on their distinct abilities to process complex datasets. For feature selection in modeling, XGBoost was chosen over LASSO because it is prone to failing to identify key nonlinear relationships between features and the target in datasets and is ineffective at prioritizing features based on their actual, non-predictive importance [17]. At the same time, XGBoost can provide an improved nonlinear boost in feature selection through its boosting procedure, eliminating features that do not significantly contribute to model predictions. Hence, this XGBoost procedure achieves a more complete and effective use of data features by eliminating unnecessary features. Additionally, this nonlinear feature boosting provides XGBoost with an advantage in improving prediction accuracy and generalizing model outcomes based on the distribution of feature importance. Subsequently, for modeling purposes, an ANN was chosen over other models, CNN-RNN, because of its distinct ability to provide multi-layer abstraction for complex input data.

### 2.5. Statistical Analysis

The entire data samples are stated as mean and standard deviation values (mean ± SD). Predictive accuracy referred to an REE prediction within ±16% of the actual value. If a predicted REE differed from the measured REE by more than 16%, then the estimation was either an underprediction or an overprediction. The predictive validity of results acquired using the various models and equations was assessed using statistical tests, including the Root Mean Square Error (RMSE), the coefficient of determination (R^2^), the intraclass correlation coefficient (ICC), the Paired Student’s *t*-Test, and the T-statistic test, as appropriate. Results are reported as mean values and Standard Deviation. A more stringent significance threshold was used, requiring a *p*-value < 0.001, to meticulously control for the increased risk of false positives associated with conducting multiple statistical tests. All data were analyzed using SPSS software version 23.0. After developing the regression equations based on the first training set, they were validated on the validation set.

## 3. Results

Based on the dataset, three non-overlapping parts were developed: for training (295, 70%), validation (64, 15%), and testing (64, 15%) of participants. This partition was vital to ensure that no fitting occurred and to assess the model’s ability to generalize to unseen data. In this case, basic learning was performed on the training data, hyperparameters were tuned on the validation data, and the test data was used exclusively for an unbiased final assessment of model performance. In addition, a five-fold cross-validation technique was used to increase statistical significance and better understand the model’s performance and accuracy. Parameters in the model were also optimized using a Grid search. These are vital steps in understanding how to balance model accuracy and generalization ability. In this case, XGBoost required proper tuning of key parameters such as learning rate, number of estimators, and max_depth. Similarly, for ANN, it was required to optimize the number of layers, learning rate, and dropout rate (see Table 2). Also, the ANN model was trained using 20 epochs with the Adam optimizer and the cross-entropy loss function. All data analyses were carried out on a Windows 10 PC with an Intel Core i7 processor and 16GB of RAM. The programming environment was based on Python 3.9.

### 3.1. Proposed Prediction Equation Through ANN

The ANN model, combined with XGBoost, helped us identify the relationships between key body composition variables and REE. As shown in Table 3, Pearson’s linear correlation coefficients indicate associations between variables extracted from XGBoost and REE. It significantly correlated with AdjBW (r  =  0.615, *p* < 0.001), FFM Index (r  =  0.603, *p* < 0.001), Age (r  =  0.527, *p* = 0.001), BMI (r  =  0.471, *p* = 0.001), and Fat Mass (r  =  0.408, *p* < 0.001). Also, the study revealed that including additional variables did not substantially improve the regression model’s predictive power. The contribution of each factor was independent and statistically significant (*p* < 0.001). Not surprisingly, AdjBW was the best predictor variable of REE.

After a careful analysis of the impact of these variables and multiple regression analysis, a predictive model for REE values in the population under consideration was developed. This is formally defined by the two equations below, where “prediction formula” denotes a relationship between the variables. The formula is derived as follows:

The first Prediction Equation (Almajwal-Abulmeaty equation_ANN1; AA_ANN1):

REE=2.47∗BMI+11.9∗AdjBW(kg)+962.5

The second Prediction Equation (Almajwal-Abulmeaty equation_ANN2; AA_ANN2):

$$REE=4.29*Age\left(years\right)+9.4*FatMass(kg)+15.71*FFMIndex(kg/m^{2})+1289.3$$

For the first REE equation, BMI (Body Mass Index) and AdjBW (Adjusted Body Weight) were used, and for the second REE equation, Age, Fat Mass, and FFM (Fat Free Mass) Index were considered. Further, the subject population (*n* = 423) was randomly divided into two groups: training and validation sets (see Table 4). Normality tests for the data distribution were also conducted using the Shapiro–Wilk and Kolmogorov–Smirnov tests.

### 3.2. Feature Ranking

**Figure 1:**
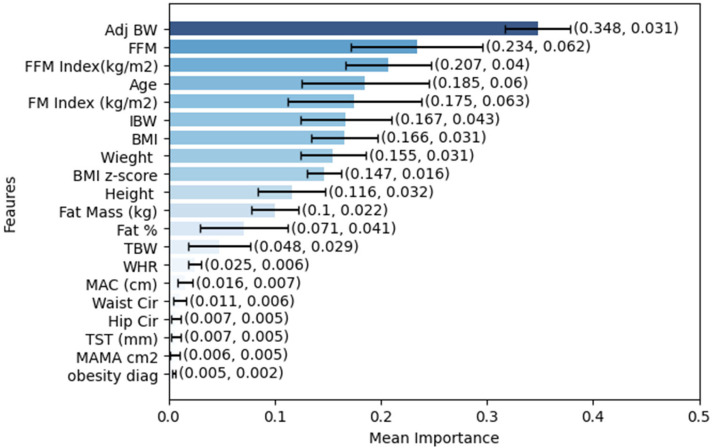
Feature ranking graph using XGBoosting with mean ± SD values.

The feature importance for the XGBoost models across the five cross-validation folds is shown in Figure 1, which presents the relative contribution of each input feature to the model’s overall predictive performance. It was observed that features such as Adjusted Body Weight (AdjBW) and Fat-Free Mass (FFM) consistently ranked among the most important, underscoring that feature rankings across datasets and model architectures depend on the composition of each training set within a k-fold cross-validation. On the contrary, features such as Obesity diagnosis, Mid-Arm Muscle Area (MAMA), Triceps Skinfold Thickness (TST), Hip, Waist, Mid-Arm Circumference (MAC), and finally, Waist-to-Hip Ratio (WHR), always ranked among the less important ones, and more importantly, presented no relative variation in their ranks among the folds. The fact that features with low predictive value were estimated to have low predictive capability suggests they could be omitted from future prediction attempts without loss of accuracy. The analysis indicates that body composition variables, specifically Age, FFM, and AdjBW, have the highest relative values across all predictions and have been confirmed as the primary predictors of REE, which is physiologically supported by their primary use in predicting REE. All models showed higher parameter importance for AdjBW, underscoring its major role in predicting REE. Nevertheless, despite the predictive role of the feature AdjBW, the relatively low overall prediction RMSE of 185 calories for the Prediction Almajwal-Abulmeaty equation_ANN1 might reflect the simplified linear relationship between AdjBW and REE.

### 3.3. Comparison of the Proposed Equations with the Published Equations

Results showed that both predictive equations (AA_ANN1 and AA_ANN2) exhibited a remarkably low prediction error compared with the REE assessed by IC and outperformed all other prediction formulas used in this research. More specifically, predictive AA_ANN1 had an RMSE of 215 and an accuracy of 66.2%, whereas AA_ANN2 had a lower RMSE of 193 and a higher accuracy of 71.4%. Such impressive validation parameters clearly indicate that the two new predictive equations accurately predicted the REE of more than 66% and 71% of the surveyed individuals, strongly supporting their validity and adequacy in young individuals of this group. Moreover, the accuracy of these new energy expenditure predictive tools and equations is of paramount importance for assessing the quality of the related evidence presented in this research (Table 5). The ICC results show that the new equations yielded moderate correlations ranging from 0.50 to 0.70. This level of agreement was comparable to that of other existing equations and even outperformed them. Therefore, we can conclude that the suggested equations produce good reliability and accuracy when determining REE over other equations from the literature.

The ANN model was trained using the top 10 features derived from XGBoost, and its predictive performance was characterized by an average prediction accuracy of 90.2% and an ICC of 0.854 (Table 5). Nevertheless, without feature refinement, the ANN model with all features yielded an even lower average prediction accuracy of 70.1%. Although the result was better than other equations and slightly lower than the prediction of AA_ANN2, the ANN model with XGBoosting was overall better than all other methods. At the same time, the prediction performance of the six other standard reference equations was substantially poorer. The lowest RMSE, 228, was obtained with the Harris–Benedict equation, achieving the best accuracy among all equations (Table 6). What was most important was that the prediction of the ANN model with the XGBoosting model was not significantly different from the actual measured REE (*p* = 0.52). Also, the prediction of the proposed equations was not significantly different from the actual (*p* = 25, *p* = 28). In contrast, the prediction of all five standard equations was significantly different from the actual REE with an extremely high significance (*p* < 0.0001) (Table 6).

**Figure 2:**
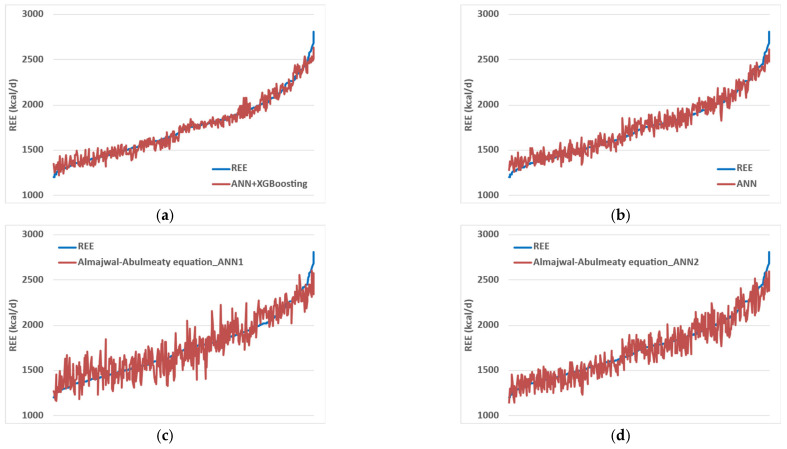

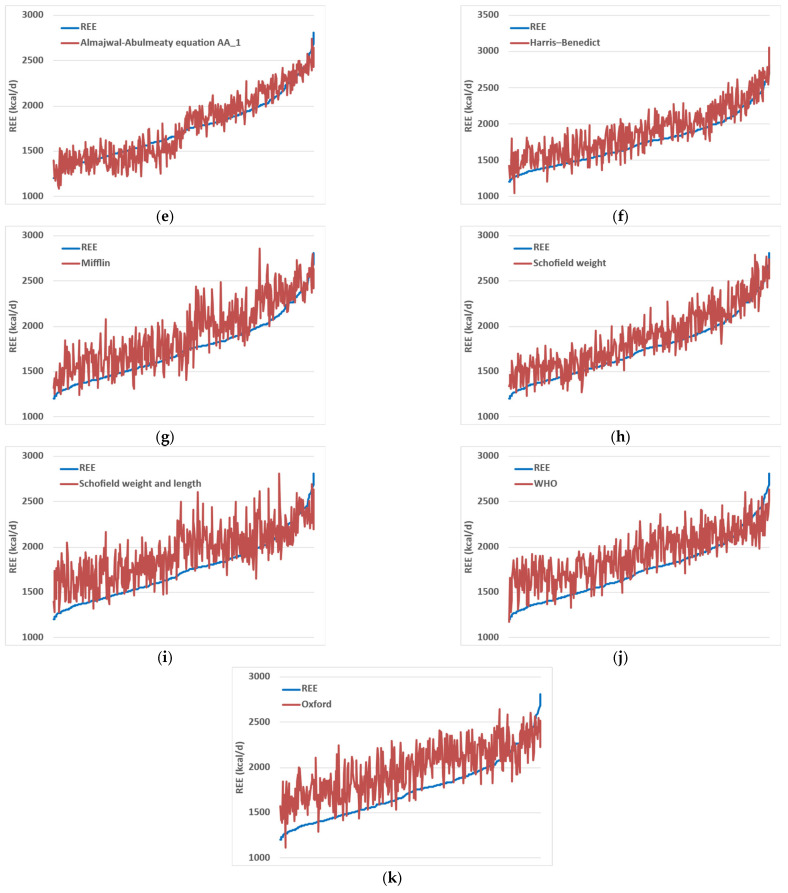
REE prediction results using (**a**) ANN + XGBoosting model, (**b**) ANN model, (**c**) Almajwal-Abulmeaty equation_ANN1, (**d**) Almajwal-Abulmeaty equation_ANN2, (**e**) equation AA_1, (**f**) Harris–Benedict, (**g**) Mifflin, (**h**) Schofield for weight, (**i**) Schofield for weight and length, (**j**) WHO, and (**k**) Oxford equations. The blue line represents the actual REE measurements, while the red line signifies the estimated fit produced by the model/equation currently being evaluated.

The final ANN model integrated with XGBoost had a strong functional approximation capability for the actual values of resting energy expenditure (REE) compared with the ANN model results. Similarly, the proposed innovative predictive equations were also aligned with the trendline of actual REE measurements. However, compared to the ANN model with the XGBoosting model, the conventional predictive equations and equations from literature tended to systematically overestimate the actual values of REE across most of the data set, except towards the latter portion of the actual values of REE, where they tended towards underestimation. Notably, the curve for the ANN model integrated with XGBoost predictive values almost overlapped with that for the actual REE measurements (Figure 2).

**Figure 3:**
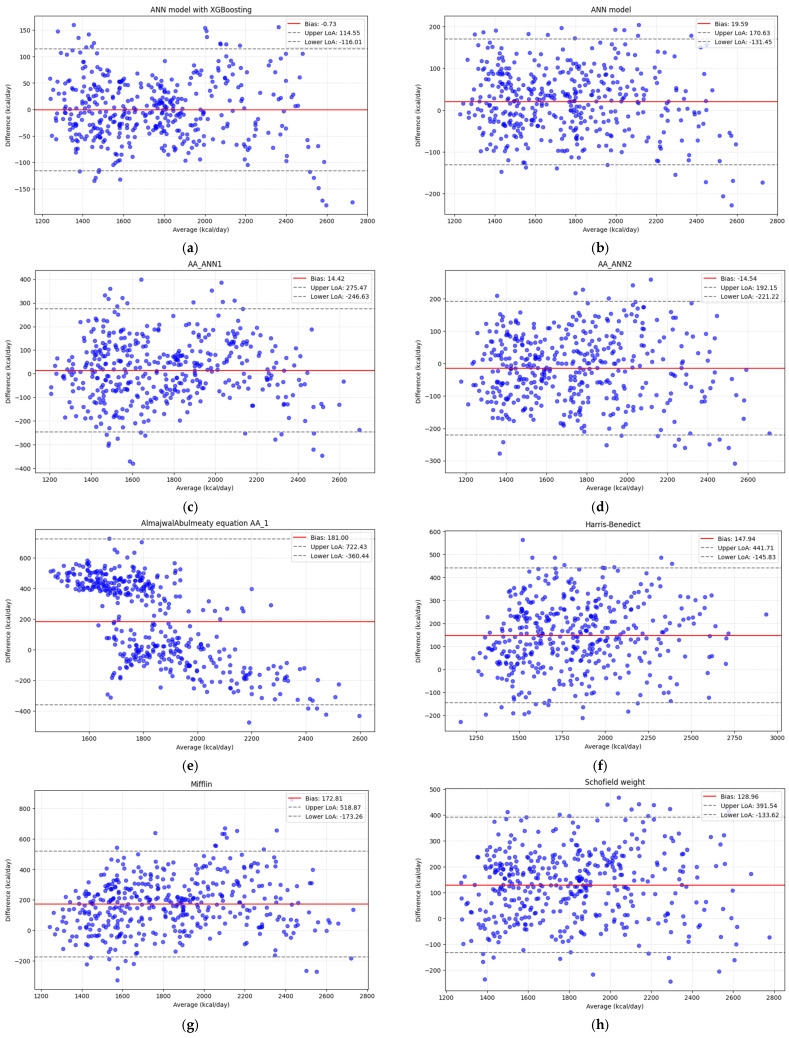

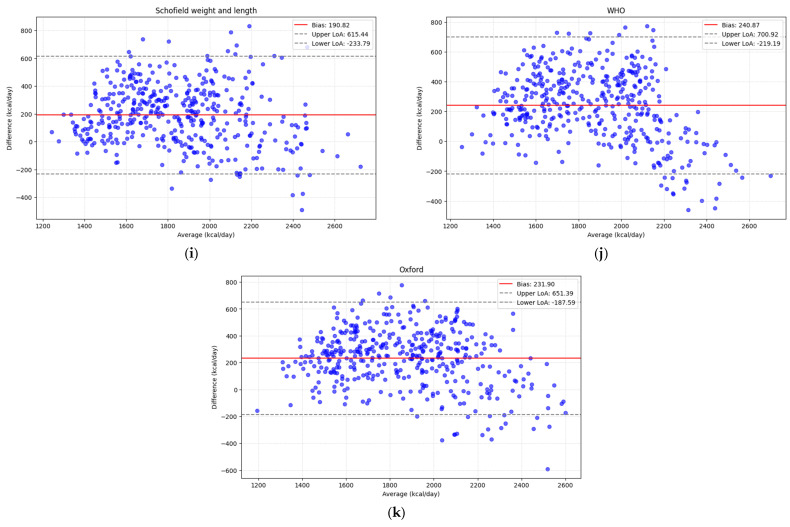
Bland–Altman plots between differences and mean predicted-measured REE using (**a**) ANN + XGBoosting model, (**b**) ANN model, and (**c**) Almajwal-Abulmeaty equation_ANN1, (**d**) Almajwal-Abulmeaty equation_ANN2, (**e**) equation AA_1 (**f**) Harris–Benedict, (**g**) Mifflin, (**h**) Schofield for weight, (**i**) Schofield for weight and length, (**j**) WHO and (**k**) Oxford equations.

The graphs in Figure 3 show the Bland–Altman comparison of the differences between actual and predicted REE, along with their means, for the ANN models and all formulas tested. Consistency was highly observed in both the developed ANN+XGBoost model and the newly suggested equations. It can be seen that the ANN+XGBoost model has been proven to be robust with negligible bias (<1 kcal) and the narrowest limit of agreement (LoA). Moreover, the two suggested equations, AA_ANN1 and AA_ANN2, have shown a narrower LoA (275-246 kcal) than other equations, indicating strong agreement with measured REE.

## 4. Discussion

In the current study, a new ANN model, along with two novel, specific predictive equations for estimating REE, was successfully developed and validated for the target adult population. Since accurate assessment of REE is of paramount importance in nutritional management and in calculating total energy needs, the developed ANN model provides an extremely valuable, highly accurate alternative for REE estimation when IC measurement is unavailable or impractical. It should finally be noted that the optimal predictive performance of these new predictive equations is achieved when they are applied to subjects whose characteristics are close to those of the subjects from whom they were derived. Accurate estimation of REE in healthy adults is particularly critical because optimizing energy intake is paramount for this population, whose overall energy needs are significantly determined by intense physical activity, which also drives energy expenditure related to normal physiological function.

The findings from this study provide important new information obtained by applying the ANN method to predict REE in adults. The predictive power of the ANN method proved to be much better, with much lower RMSE values than the newly derived equations used in this study, as well as the six previously validated equations used for comparison in this study on REE prediction in adults. The output values derived using the ANN-based method showed very close correlations with the measured REE values. The applicability and relevance of the ANN-based prediction method gains importance particularly because the existing prediction equations, such as the widely used Harris–Benedict equation [3] published in the early 1920s, may actually have been generated utilizing population pools that had different nutritional practices, lifestyles that could potentially be less sedentary, and body compositions that could potentially contain higher proportions of FFM, reflecting the general trend for the last century toward increased sizes for all dimensions, especially reflecting the worldwide trend toward increased adiposity levels for the general population. The application validity for the methods and equations developed eight decades ago may actually harbor systemic errors due to the observed population alterations in recent years, mainly due to the overall increase in dimensions and potential alterations in FFM proportions, reflecting the overall trend toward higher proportions for all aspects, reflecting the population alterations for the last century.

This original study used linear regression analysis to derive and validate two new predictive equations for REE in the adult population under study. While it is recognized that predictions cannot replace the values of REE, as established by IC, our large, relatively homogeneous population leads us to believe that our new equations may still be used on similarly composed adults. The first AA_ANN1 equation, based on readily available anthropometric parameters such as AdjBW and BMI, is easy for dietitians and medical practitioners to use and requires only access to a balance and a stadiometer. The second AA_ANN2 equation, based on body composition parameters (Fat Mass, FFM index) together with age, may be viewed as more population-specific, since the FFM index is widely accepted as the principal predictor of REE values across almost all populations [18,19]. The second, however, requires more time and specialized equipment from the person measuring the subjects’ body composition parameters. Also, the widely documented reduction in REE with age is, in turn, due to alterations in FFM [20,21]. Body weight is also a significant factor in determining REE, considering the composition of body tissues such as muscle, fat-free mass, and fat mass, which are all involved in the process of thermogenesis [22]. Along the same lines, height is also a significant factor in determining REE, as taller people tend to have larger organ systems, thereby requiring more energy [22]. Furthermore, BMI and AdjBW, both indicative of body mass, confirm the association between body composition and REE [23,24]. These significant factors, including age, fat mass, FFM index, AdjBW, and BMI, were thus incorporated into the newly developed predictive equations.

Our new equations were rigorously validated on a sample of 423 subjects, achieving high predictive performance of 66.2% and 71.4% for the AA_ANN 1 and 2, respectively, and exhibiting very small RMSE and standard deviation of the differences between the predicted and actual REE values in our validation group. Jalilpiran et al. [25] noted that equations predicting REE tend to overestimate REE in people with lower REE and underestimate it in people with higher REE. Therefore, recognizing this bias, even in the most commonly used equations, can improve decision-making regarding information gathering, especially for people with low or high REE levels. This is because the phenomenon reflects the inherent disadvantage of using mean prediction values, which can cause overestimation in people with low REE and underestimation in people with high REE, leading to inappropriate and misleading information gathering. Moreover, some previous research has shown that for obese populations, equations that use body composition measurements have not significantly improved predictive accuracy compared with those that use only age, height, and weight [26]. However, a study by Achamrah et al. [27] reported that Müller equation-based bioelectrical impedance analysis improved the accuracy of REE estimation compared with methods that do not use body composition measurements (67.1% vs. 60.3%). This study suggests that the FFM index is a much better explanatory variable for REE values than body weight. While the FFM index actually reflects differences in REE, it should nevertheless not be forgotten that genetic factors play a role in the existing variations in REE between populations, hence the fact that equations based on body composition parameters are naturally population-specific [28]. Moreover, while age and physical activity have proven significantly influential on FFM index values, the physiological underpinnings of body composition and physical activity are clearly distinct from, though similarly pertinent to, the underlying task of REE prediction.

This study represents a first in challenging established REE prediction methodologies in adults, not only by the ANN approach but also with equation development. This is also a strength of the study, as REE was measured with great care in a large cohort of adults. There are, however, a few methodological considerations that raise limitations. Specifically, concerns may arise because the final calculations derived from IC equipment at times rely on the same underlying prediction equations being challenged, and thus may introduce a degree of circularity or bias. The evaluation of body composition by BIA may show minor differences across various manufacturers. Thus, more sophisticated and objective measurement techniques would likely provide greater resolution and reliability for body composition metrics.

Owing to the absence of existing datasets that aligned with both the specific variables of interest and the designated geographic focus on Saudi Arabia, this study used a limited dataset with a small number of participants. The future study will focus on enhancing and enriching the dataset in terms of the number of participants and the region.

## 5. Conclusions

When IC is unavailable, the best prediction of REE was obtained using ANN approaches, especially when combined with XGBoost, and with a population-specific sample. The full ANN model achieves a prediction accuracy of 90.2%, while the simplified ANN equations achieve 66.2% and 71.4%, respectively. Additionally, future external validation in an independent cohort is essential before these equations can be recommended for clinical use. 

## Figures and Tables

**Table 1:** Anthropometric data and major metabolic parameters with mean and SD.

		Male (Mean ± SD) (*n* = 208)			Female (Mean ± SD) (*n* = 215)		
Variable	Total Mean ± SD (*n* = 423)	Normal Weight (*n* = 59)	Over Weight (*n* = 80)	Obese (*n* = 69)	Normal Weight (*n* = 75)	Over Weight (*n* = 76)	Obese (*n* = 64)
Age	28.12 ± 8.01	23.6 ± 5.6	26.4 ± 8.7	25.0 ± 6.8	28.2 ± 5.3	31.5 ± 8.1	33.6 ± 81
Height	164.39 ± 8.52	170.9 ± 5.9	170.2 ± 5.4	171.5 ± 6.4	158.0 ± 5.5	159.0 ± 53	157.4 ± 5.6
Weight	77.3 ± 19.06	65.5 ± 6.6	79.5 ± 6.5	108.5 ± 14.6	56.3 ± 5.8	69.4 ± 6.2	85.7 ± 12.1
BMI	28.49 ± 6.13	22.4 ± 1.8	27.4 ± 1.4	36.9 ± 5.0	22.5 ± 1.7	27.4 ± 1.4	34.6 ± 4.3
IBW	57.81 ± 9.25	65.4 ± 6.4	64.5 ± 5.8	66.0 ± 6.9	50.4 ± 4.9	51.4 ± 4.7	49.9 ± 5.1
Adj BW	62.68 ± 10.12	65.4 ± 5.9	68.3 ± 5.7	76.6 ± 7.2	51.9 ± 4.8	55.9 ± 4.9	58.9 ± 5.9
Waist Cir	87.8 ± 14.91	76.8 ± 7.8	89.7 ± 8.1	111.4 ± 10.5	73.1 ± 6.3	82.4 ± 6.6	93.8 ± 8.8
Hip Cir	104.86 ± 13.57	92.7 ± 5.6	102.8 ± 12.7	120.4 ± 10.5	93.8 ± 6.9	103.6 ± 6.1	116.5 ± 9.6
WHR	0.84 ± 0.09	0.83 ± 0.1	0.88 ± 0.1	0.93 ± 01	0.78 ± 0.1	0.80 ± 0.1	0.81 ± 0.1
MAC (cm)	30.42 ± 4.54	26.3 ± 2.4	28.8 ± 2.2	33.5 ± 4.4	27.1 ± 2.4	31.1 ± 2.6	36.0 ± 4.1
TST (mm)	27.17 ± 8.9	17.1 ± 6.7	25.4 ± 6.6	34.3 ± 6.3	22.3 ± 6.3	29.5 ± 6.7	34.0 ± 7.3
MAMA cm^2^	39.13 ± 13.27	35.5 ± 10.8	35.0 ± 8.0	42.5 ± 14.7	32.9 ± 8.5	38.4 ± 8.9	52.2 ± 17.7
Fat %	31.57 ± 9.4	17.6 ± 5.1	25.1 ± 4.0	34.8 ± 5.2	30.0 ± 4.6	37.6 ± 3.2	43.8 ± 6.0
Fat Mass (kg)	24.95 ± 11.32	11.6 ± 4.0	19.9 ± 3.8	38.0 ± 10.0	17.1 ± 3.9	26.2 ± 4.0	37.3 ± 7.9
FM Index (kg/m^2^)	9.31 ± 4.25	4.0 ± 1.3	6.9 ± 1.3	13.0 ± 3.5	6.8 ± 1.4	10.3 ± 1.3	15.0 ± 3.0
FFM	52.12 ± 11.57	53.4 ± 5.2	59.3 ± 5.2	70.0 ± 7.7	39.2 ± 3.0	43.3 ± 3.3	48.4 ± 5.1
FFM Index (kg/m^2^)	19.11 ± 2.99	18.3 ± 1.2	20.4 ± 1.1	23.8 ± 2.4	15.7 ± 0.9	17.1 ± 0.8	19.5 ± 1.7
TBW	46.24 ± 10.1	57.0 ± 8.5	52.6 ± 5.4	49.1 ± 5.0	40.8 ± 11.8	39.9 ± 7.6	39.3 ± 4.4
RQ	0.76 ± 0.09	0.79 ± 0.1	0.79 ± 0.1	0.81 ± 01	0.72 ± 0.1	0.71 ± 0.1	0.72 ± 0.1
VO_2_	258 ± 56.07	258.5 ± 33.7	283.1 ± 42.2	331.5 ± 50.0	204.6 ± 24.1	227.1 ± 34.5	246.3 ± 39.6
VCO_2_	196.29 ± 52.8	203.0 ± 29.1	222.9 ± 40.0	268.2 ± 47.2	146.7 ± 19.6	163.4 ± 27.9	176.5 ± 29.5

**Table 2:** Hyperparameters used for XGBoost and ANN models.

Parameter	Values Range	Selected Value
For the XGBoost model		
Learning rate	0.01–1.0	0.1
Estimators	50–500	100
max_depth	3–6	5
For the ANN model		
Layers	2–8	3
Learning Rate	0.01, 0.005, 0.001, 0.0005, 0.0001	0.001
Dropout Rate	0.2–0.7	0.4

**Table 3:** Linear Correlation between REE and variables extracted from XGBoost.

Particular	r
Adj BW	0.615 **
FFM Index(kg/m^2^)	0.603 **
Age	0.527 **
BMI	0.471 **
Fat Mass (kg)	0.408 **
FM Index (kg/m^2^)	0.281 *
IBW	0.394 *
FFM	0.168
Weight	0.135
BMI z-score	0.114
Height	0.306 *
Fat %	0.187
TBW	0.216 *
WHR	0.375 *
MAC (cm)	0.293 *
Waist Cir	0.196 *
Hip Cir	0.107
TST (mm)	0.124
MAMA cm^2^	0.241 *

** correlation is significant below 0.001. * correlation is significant below 0.01.

**Table 4:** Characteristics of training and validation sets with mean and SD.

Particular	Mean ± SD (*n* = 295)	Mean ± SD (*n* = 128)
Adj BW	62.48 ± 9.92	62.48 ± 9.92
FFM Index(kg/m^2^)	18.71 ± 2.59	18.91 ± 2.79
Age	28.42 ± 8.31	27.92 ± 7.81
BMI	28.09 ± 5.73	28.09 ± 5.73
Fat Mass (kg)	24.45 ± 10.82	24.95 ± 11.32
FM Index (kg/m^2^)	9.71 ± 4.65	9.01 ± 3.95
IBW	57.31 ± 8.75	58.21 ± 9.65
FFM	52.32 ± 11.77	52.12 ± 11.57
Weight	76.9 ± 18.66	76.9 ± 18.66
BMI z-score	−0.5 ± 0.5	0.3 ± 1.3
Height	163.89 ± 8.02	164.69 ± 8.82
Fat %	31.37 ± 9.2	31.27 ± 9.1
TBW	46.14 ± 10	45.84 ± 9.7
WHR	1.04 ± 0.29	0.84 ± 0.09
MAC (cm)	30.12 ± 4.24	30.72 ± 4.84
Waist Cir	87.5 ± 14.61	87.6 ± 14.71
Hip Cir	104.96 ± 13.67	105.16 ± 13.87
TST (mm)	27.47 ± 9.2	27.57 ± 9.3
MAMA cm^2^	39.53 ± 13.67	38.73 ± 12.87

**Table 5:** Comparison of results of predicted REE with the equations developed in this study (including Almajwal-Abulmeaty equation_ANN1 and 2) through mean and SD, along with the RMSE, R^2^, T-statistics, *t*-Test, accuracy, and ICC.

Method	Mean (kcal/d)	SD	RMSE	R^2^	T-Statistics	*t*-Test	Accurate Prediction (%)	Under Prediction (%)	Over Prediction (%)	ICC
REE Measured	1749	329								
AA_ANN1	1774	228	215	0.539	8.32	0.25	66.2	15.5	18.3	0.542 ✦ [0.519, 0.593]
AA_ANN2	1742	194	193	0.562	−7.51	0.28	71.4	22.4	6.2	0.583 ✦ [0.529, 0.634]
ANN model	1791	219	217	0.651	6.46	0.27	70.1	18.3	11.6	0.626 ✦ [0.529, 0.675]
ANN model with XGBoosting	1745	163	179	0.884	−1.07	0.52	90.2	2.5	7.3	0.854 ✦ [0.829, 0.893]

REE is resting energy expenditure; SD is standard deviation; RMSE is Root Mean Square Error; AA_ANN1 is Almajwal-Abulmeaty equation_Artificial Neural Network 1; AA_ANN2 is Almajwal-Abulmeaty equation_Artificial Neural Network 2; ANN model is Artificial Neural Network model; ANN model with XGBoosting is an Artificial Neural Network model with Extreme Gradient Boosting. ICC: Intraclass correlation coefficient (ICC, with 95% confidence interval): ✦ > 0.70: Good reliability, ✦ 0.50–0.70: Moderate reliability, ✦ < 0.50: Poor reliability.

**Table 6:** Comparison of results of predicted REE with state-of-the-art methods from literature and the best method from this study through mean and SD, along with the RMSE, R^2^, T-statistics, *t*-Test, accuracy, and ICC.

Method	Mean (kcal/d)	SD	RMSE	R^2^	T-Statistics	*t*-Test	Accurate Prediction (%)	Under Prediction (%)	Over Prediction (%)	ICC
REE Measured	1749	329								
Mifflin [6]	1906	257	231	0.219	20.45	<0.001	58.2	17.4	24.4	0.416 ✦ [0.271–0.502]
Harris–Benedict [3]	1910	392	228	0.135	23.19	<0.001	59.1	23.5	17.4	0.378 ✦ [0.194–0.459]
Schofield’s weight and length [13]	1893	289	245	0.216	26.37	<0.001	54.3	27.2	18.5	0.328 ✦ [0.295–0.431]
Schofield weight [13]	1801	315	325	0.208	27.28	<0.001	58.1	19.5	22.4	0.295 ✦ [0.204–0.365]
WHO [15]	2088	306	329	0.247	21.04	<0.001	50.6	34.4	15	0.283 ✦ [0.155–0.324]
Oxford [14]	2152	353	318	0.245	19.26	<0.001	50.1	28.7	21.2	0.247 ✦ [0.104–0.271]
AA_1 [7]	1834	214	251	0.549	−5.49	0.19	64.3	20.3	15.4	0.531 ✦ [0.502–0.531]
ANN model with XGBoosting	1745	163	179	0.884	−1.07	0.52	90.2	2.5	7.3	0.854 ✦ [0.829, 0.893]

REE is resting energy expenditure; SD is standard deviation; RMSE is Root Mean Square Error; AA_1 is Almajwal-Abulmeaty equation (AA_1); ANN model with XGBoosting is an Artificial Neural Network model with Extreme Gradient Boosting. ICC: Intraclass correlation coefficient (ICC, with 95% confidence interval): ✦ > 0.70: Good reliability, ✦ 0.50–0.70: Moderate reliability, ✦ < 0.50: Poor reliability.

## Data Availability

Data supporting this work are available on reasonable request from the corresponding author.

## Abbreviations

The following abbreviations are used in this manuscript:

AA	Almajwal and Abulmeaty
ANN	Artificial neural network
AdjBW	Adjusted Body Weight
AI	Artificial intelligence
BMI	Body Mass Index
BIA	Bioelectric impedance analysis
CNN-RNN	Convolutional Neural Network-Recurrent Neural Network
DNN	Deep neural network
XGBoost	Extreme Gradient Boosting
FFMI	Fat Free Mass Index
FMI	Fat mass index
GBM	Gradient Boosting Machine
LoA	Limit of Agreement
MAMA	Mid-arm muscle area
MAC	Mid-arm circumference
NCDs	Non-communicable diseases
IBW	Ideal body weight
IC	Indirect Calorimetry
REE	Resting energy expenditure
RMSE	Root Mean Square Error
RQ	Respiratory Quotient
ReLU	Rectified Linear Unit
SHAP	SHapley Additive exPlanations
TST	Triceps skinfold thickness
TBW	Total body water
WHR	Waist-to-Hip Ratio
VO_2_	Volumes of respiratory oxygen
VCO_2_	Volumes of respiratory carbon dioxide
